# Genomics insight on passion fruit viral disease complexity

**DOI:** 10.1128/spectrum.00344-25

**Published:** 2025-08-29

**Authors:** Florence Munguti, Evans N. Nyaboga, Timothy Holton, Jan Kreuze, Solomon Maina

**Affiliations:** 1 Kenya Plant Health Inspectorate Service, Nairobi, Kenya; 2 Department of Biochemistry, University of Nairobi 107854, Nairobi, Kenya; 3 Biosciences eastern and central Africa-International Livestock Research Institute 735343, Nairobi, Kenya; 4 Health and Quarantine Unit, International Potato Center 54718, Lima District, Peru; 5 New South Wales Department of Primary Industries and Regional Development, Agriculture and Biosecurity, Elizabeth Macarthur Agricultural Institute 153388 , Menangle, New South Wales, Australia; USDA-ARS San Joaquin Valley, Parlier, California, USA

**Keywords:** passion fruit woodiness disease, metagenomics, viruses

## Abstract

Passion fruit viral diseases pose a significant threat to Kenya’s passion fruit industry. To unravel the complexity of these diseases, comprehensive virus surveys were conducted across major passion fruit-growing counties. Passion fruit woodiness disease symptoms, like fruit hardening, chlorotic mottling, and leaf distortion, were prevalent. The study unveiled the first 23 complete genomes of Ugandan passiflora virus (UPV) and two East Asian passiflora distortion virus (EAPDV) in Kenya. UPV showed 99% nucleotide (nt) match to a UPV genome from Uganda and 66% nt identity match to EAPDV. In addition, UPV variants and two partial passion fruit green spot virus sequences and partial (passiflora emaravirus) segment RNA1-5 (novel allexivirus and an emaravirus, respectively) were detected. Phylogenetic analysis revealed distinct lineages (I–III), indicating potential multiple introductions into Kenya. Recombination analysis detected no significant breakpoints. However, the study proposed the renaming of EAPDV to passiflora distortion virus (PDV) and UPV to passiflora virus (PV) for neutral nomenclature, without geographical association. Additionally, the study highlighted the role of coinfections in symptom expression, suggesting a potential synergistic relationship between PV, PDV, and other viruses. The results recommend stringent management strategies and enhanced surveillance to mitigate the economic impact of these viruses on the Kenyan passion fruit industry. The findings from this study underscore the need to strengthen nursery certification programs and pest diagnostic protocols in Kenya. Additionally, enhanced pest surveillance and import regulations are critical to preventing the introduction and spread of emerging plant viral diseases, thereby safeguarding the country’s horticultural productivity and biosecurity. To our knowledge, this is the first comprehensive study of viral diseases of passion fruit in Kenya.

**IMPORTANCE:**

This study presents the first comprehensive survey of viral pathogens affecting passion fruit in Kenya, identifying Ugandan passiflora virus (UPV) and East Asian passiflora distortion virus (EAPDV) as major contributors. Through genomic sequencing, 23 complete genomes of UPV and two of EAPDV were characterized, revealing a 99% nucleotide (nt) similarity between UPV strains from Uganda and Kenya, and 66% nt match with EAPDV. Phylogenetic analysis identified distinct lineages, suggesting possible multiple viral introductions in Kenya. The study also highlights potential synergistic coinfections between UPV, EAPDV, and other viruses, leading to more severe disease symptoms. In light of these findings, the study proposes renaming EAPDV as passiflora distortion virus and UPV as passiflora virus for a more neutral name classification. The research underscores the urgent need for enhanced surveillance, stringent phytosanitary measures, and improved management strategies to mitigate the threat of viral diseases, to safeguard the Kenyan passion fruit industry, and elsewhere.

## INTRODUCTION

Passion fruit (*Passiflora edulis*) is a dicotyledonous perennial plant with shallow roots and woody vines that climb by use of tendrils. It belongs to the family *Passifloraceae* with a wide genetic base of approximately 525 species (1). There are about 60 species of passion fruit producing edible fruits, and the most important include the yellow passion fruit (*Passiflora edulis var. flavicarpa*) and the purple passion fruit (*Passiflora edulis var. purplar*) (2). Passion fruit is an important crop due to its aromatic edible fruits, medicinal properties, and ornamental use. It is an important source of minerals and vitamins, phytoconstituents, and phenolic compounds (3, 4). Passion fruit is thought to have originated from South America (5, 6). The crop was introduced into England in 1810 and later spread to Australia, Hawaii, and South Africa in 1880 and to Kenya in 1920. Today, passion fruit is widely cultivated in nearly all tropical and subtropical regions in the world (7).

The production potential of passion fruit in Kenya is estimated at 24–30 ton ha^−1^ (8, 9). However, Kenya’s average yield is low (8 ton ha^−1^) mainly due to a significant number of pests and diseases (caused by bacteria, fungi, and viruses) and inadequate clean planting materials (9–11). Yield losses of 80%–100% have been attributed mainly to multiple occurrences of diseases (10, 12, 13), which is contributed by lack of adequate certified disease-free planting materials (14). These constraints have also led to the reduction of the lifespan of the plants in the field from 7 years to an average of 1–2 years (9, 15).

Viruses are a major cause of diseases in passion fruit production and have posed a significant threat to fruit yields and quality (16). Currently, more than 25 different viruses belonging to the genera *Potyvirus*, *Cucumovirus*, *Begomovirus*, *Tymovirus*, *Cilevirus*, *Carlavirus*, *Tobamovirus*, and *Nepovirus* have been identified and characterized in passion fruit plants (7, 17–27). Of these, the cowpea aphid-borne mosaic virus (CABMV, genus *Potyvirus*) and Ugandan passiflora virus (UPV, genus *Potyvirus*) in East Africa have been reported to be the causal agents of woodiness disease, the most limiting disease in passion fruit production (20, 21, 28). This viral disease is known to cause hardening, thick-skinned, small-sized fruits with a diminished pulp. Other viral pathogens from other parts of the world have been designated as putative etiological agents of woodiness disease, including passion fruit woodiness virus (PWV) in Australia and Brazil (29), East Asian passiflora virus (EAPV) in Japan (29), and South African passiflora virus in South Africa (30), which was later found to be similar to CABMV (22, 31). Despite reports of passion fruit woodiness disease (PWD)-complex being the most important constraint in Kenya passion fruit production areas (28, 32, 33), limited studies have been done to characterize the virus population implicated with PWD. In Kenya, the most recently identified virus infecting passion fruit is the *Potyvirus* CABMV (21, 28).

Given that a broad diversity of passion fruit-infecting viruses causing PWD has been found in other parts of the world (29, 34, 35), more etiological agents of PWD and passion fruit virome complexity remain to be discovered in East Africa. In addition, the lack of holistic information on viruses infecting passion fruit in Kenya hinders the development of diagnostic tools, effective control measures, such as cross-protection and the creation of broad-spectrum virus-resistant passion fruit varieties through conventional breeding. High-throughput sequencing (HTS) has immense potential in identifying unknown plant viruses, diverse strains, or new variants (36–39). This study explored: (i) the discovery and understanding of viruses causing passion fruit woodiness disease complex in major passion fruit growing regions of Kenya, (ii) providing first complete genomes of passion fruit related virome in Kenya and investigating whether any genetic relationship exists with other viruses in global database, and (iii) investigating genetic recombination process within the newly discovered viruses and its role in driving virulence.

## MATERIALS AND METHODS

### Collection of passion fruit leaf samples

**Fig 1:**
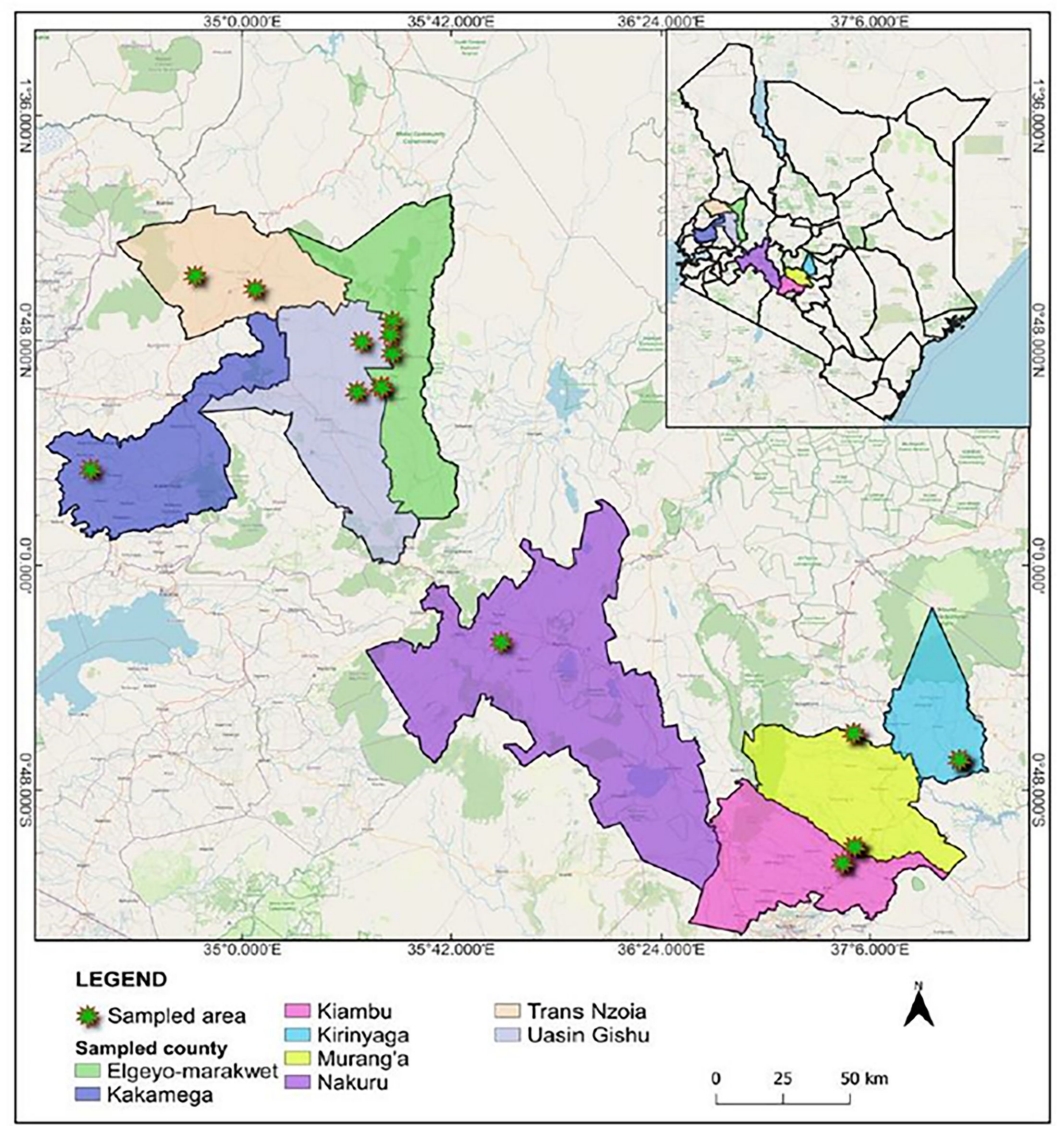
Map of Kenya showing the geographical field locations (counties) where all the passion fruit leaf samples were collected. The map was produced using open source Quantum GIS, popularly QGIS.

Passion fruit vines showing virus-like symptoms and non-symptomatic plants were sampled from smallholder farms in major passion fruit growing areas in Kenya between August and November 2014 (Table 1; Fig. 1). Five plants were sampled per field by collecting three leaf samples in each plant (from upper, middle, and lower leaves within a vine). The samples from each plant were bulked for virus testing. A total of 22 bulked samples were collected from Rift Valley (Nakuru [1], *Trans* Nzoia [3], Elgeyo Marakwet [5], and Uasin Gishu [2]), Central (Kirinyaga [3], Murang’a [2], and Kiambu [5]), and Western (Kakamega [1]) regions in Kenya, placed and sealed in zip-lock bags containing silica gel, and preserved for at least one month prior to RNA extractions.

**TABLE 1:** Cultivar type, symptoms, and geographical information of passion fruit leaf samples that were used to uncover passion fruit virome using HTS in Kenya^*a*^

Sample no.	HTS no.	Cultivar	Fruit type	Viral-like disease symptoms	County	Region
K1	1_S1_L001	KPF 11	Yellow	Symptomatic	Kirinyaga	Central
K2	2_S2_L001	KPF 11	Yellow	Symptomatic	Kirinyaga	Central
K3	3_S3_L001	KPF4	Yellow	Symptomatic	Kirinyaga	Central
K4	4_S4_L001	KPF 12	Yellow	Symptomatic	Kiambu	Central
K5	5_S5_L001	KPF 4	Yellow	Symptomatic	Kiambu	Central
K6	6_S6_L001	KPF4	Yellow	Asymptomatic	Murang’a	Central
K7	7_S7_L001	Unknown	Hardy shell	Symptomatic	Murang’a	Central
K8	8_S8_L001	Unknown	Purple	Symptomatic	Kiambu	Central
K9	9_S9_L001	Unknown	Sweet yellow	Symptomatic	Kiambu	Central
K10	10_S10_L001	Unknown	Sweet yellow	Symptomatic	Kiambu	Central
K11	11_S11_L001	Unknown	Purple	Symptomatic	Nakuru	Rift Valley
K12	12_S12_L001	Unknown	Purple	Symptomatic	Uasin Gishu	Rift Valley
K13	13_S13_L001	Unknown	Purple	Symptomatic	Uasin Gishu	Rift Valley
K14	14_S14_L001	Unknown	Yellow	Symptomatic	Elgeyo Marakwet	Rift Valley
K15	15_S15_L001	Unknown	Purple	Symptomatic	Elgeyo Marakwet	Rift Valley
K16	16_S16_L001	Unknown	Purple	Symptomatic	Elgeyo Marakwet	Rift Valley
K17	17_S17_L001	Unknown	Purple	Symptomatic	Elgeyo Marakwet	Rift Valley
K18	18_S18_L001	Unknown	Purple	Symptomatic	Elgeyo Marakwet	Rift Valley
K19	19_S19_L001	Unknown	Grafted	Symptomatic	Trans Nzoia	Rift Valley
K20	20_S20_L001	Unknown	Grafted	Symptomatic	Trans Nzoia	Rift Valley
K21	21_S21_L001	Unknown	Grafted	Symptomatic	Trans Nzoia	Rift Valley
K22	22_S22_L001	Unknown	Grafted	Symptomatic	Kakamega	Western

^*a*^ The samples were collected between August and November 2014.

### RNA extraction and RT-PCR

The RNA was extracted from the 22 passion fruit leaf samples preserved in silica gel using TRIzol reagent (Sigma-Aldrich, St. Louis, MO, USA) as described in ( 21), followed by DNase treatment using DNA-free Kit (Invitrogen) with incubation at 37°C for 30 min. The RNA quality control was performed using a Qubit (Invitrogen), and the integrity was further confirmed using RNA screentape (TapeStation 2200; Agilent Technologies). The extracted RNA was stored at −80°C awaiting library preparation. Total RNA extracts were also used as templates for RT-PCR using the Superscript One-Step RT-PCR System with Platinum (Invitrogen). Several PCR parameters, such as annealing temperature, extension, and cycling times, were investigated to determine the optimal RT-PCR assay with each primer PV_F-AGATCGACGCAGGTAAAGATAAG and PV_R-CAATTGGCTTCAACGGGTATTC and PDV_F-GTGGCTGGATGTTATGGGAATA and PDV_R-CTCTTTCTTAGGGTGTCGGAATAG having a concentration of 10 pmol/µL in a 20 µL reaction. Two microliter each from RNase/DNase-free water and RNA from a chickpea plant were included as negative controls. Optimum cycling conditions were determined as follows: 50°C for 30 min for reverse transcription, 95°C for 15 min, followed by 35 cycles at 95°C for 30 s, 58°C for 40 s, and 72°C for 45 s, with a final extension at 72°C for 10 min. The amplified PCR products were confirmed by agarose gel electrophoresis followed by SYBR safe staining.

### High-throughput sequencing

The RNA extracted from passion fruit leaf samples was used for library preparation, using the TruSeq Stranded Total RNA Sample Preparation kit with Ribozero Plant (Illumina, San Diego, CA). This involved subjecting the RNA to depleting ribosomal RNA using rRNA binding beads. The RNA was then fragmented and primed with random hexamers. First strand cDNA was synthesized using a mixture of Superscript II and Actinomycin D to allow RNA-dependent synthesis and inhibit DNA-dependent synthesis, respectively. The second strand cDNA synthesis was done using dUTP instead of dTTP to create stranded cDNA. The libraries were then adenylated at the 3′ end using set A and B adaptors. The cDNA fragment was enriched by 15 cycles of PCR. The final size and concentration of each library were verified using Qubit and D1000 ScreenTape (TapeStation 2200; Agilent Technologies). Ten nanomolar library pools were prepared by mixing the libraries to achieve an equal molar concentration. The 22 libraries were pooled and normalized to 10 pM, and a 1% PhiX v3 spike was included. Sequencing was conducted using MiSeq cycle 2 × 251 v3 kit (Illumina) to generate paired-end reads at Biosciences eastern and central Africa-International Livestock Research Institute (BecA-ILRI), Nairobi, Kenya.

### Sequence analysis

The RNA-Seq raw reads were first trimmed using Trim Galore (40), with minimum sequence length set to 50 bp and minimum required adapter overlap (stringency) set to 1 bp. *De novo* assembly was performed using the metaSPAdes version 3.13.0 genome assembler (41) with default settings. In addition, a second assembler, CLC Genomics Workbench 20 (CLCGW; CLC bio, Qiagen, Redwood City, CA) was used with the quality scores limit set to 0.01, maximum number of ambiguities to two, and removing any reads with <50 nt. *De novo* assembly was performed using CLCGW with settings of automatic word size, automatic bubble size, minimum contig length 800, mismatch cost two, insertion cost three, deletion cost three, length fraction 0.5, and similarity fraction 0.9 (42–44). Using CLCGW, contigs were sorted by length, examined individually, and then subjected to NCBI BLAST and PASC sequence comparisons (45, 46). In addition, trimmed reads from CLCGW were also imported into Geneious Prime (Biomatters Ltd, Auckland, New Zealand) (47) for reference mapping using a minimum overlap of 10%, a minimum overlap identity of 80%, allowing gaps of 10%, and fine-tuning set to iterate up to 10 times. Virus coding regions were improved by aligning nt sequences to the aligned deduced amino acid sequences using MUSCLE available in Geneious. Open reading frames were predicted, and annotations were performed with transfer annotation selected and similarity set at 80%, while other settings were left as default. The normalized depth was calculated using: (depth/total number of reads) × 1,000,000.

### Phylogenetic and recombination analysis

All available complete genome sequences of potyviruses reported to infect passion fruit were downloaded manually from NCBI and aligned with the *Potyvirus* consensus sequences found in this study using the Muscle algorithm as implemented in MEGA-X (48). Pairwise sequence distances and the optimal evolutionary model were then calculated for each alignment using MEGA-X. The optimal model for each alignment was then applied for phylogenetic analysis using the maximum likelihood method in MEGA-X, with a 50% cut-off bootstrap value. Recombination analysis was performed using RDP version 4.97 (49) to detect putative recombination break points between 21 UPV and CABMV sequences. Any detected recombination signals flagged by RDP 4.97 as potentially arising through evolutionary processes other than recombination, showing low bootstrap support values, and displaying inconsistent signals across methods were disregarded. Default parameters were used for the seven recombination detection methods implemented: RDP5.2 (50), GENECONV (51), Bootscan (52), MaxChi (53), Chimaera (54), 3Seq (55), and SiScan (56). To avoid false positives, only potential recombination events with an associated Bonferroni-corrected *P* value < 0.05 of more than four recombination detection methods were considered credible evidence of recombination (57). The BURT method of reference 49 was used to infer the locations and 95% confidence intervals of break point locations. Recombination breakpoint locations and origins of sequence regions potentially transferred during recombination were verified individually using the phylogenetic tools implemented in RDP 4.97.

## RESULTS

### Virus identification

**Fig 2:**
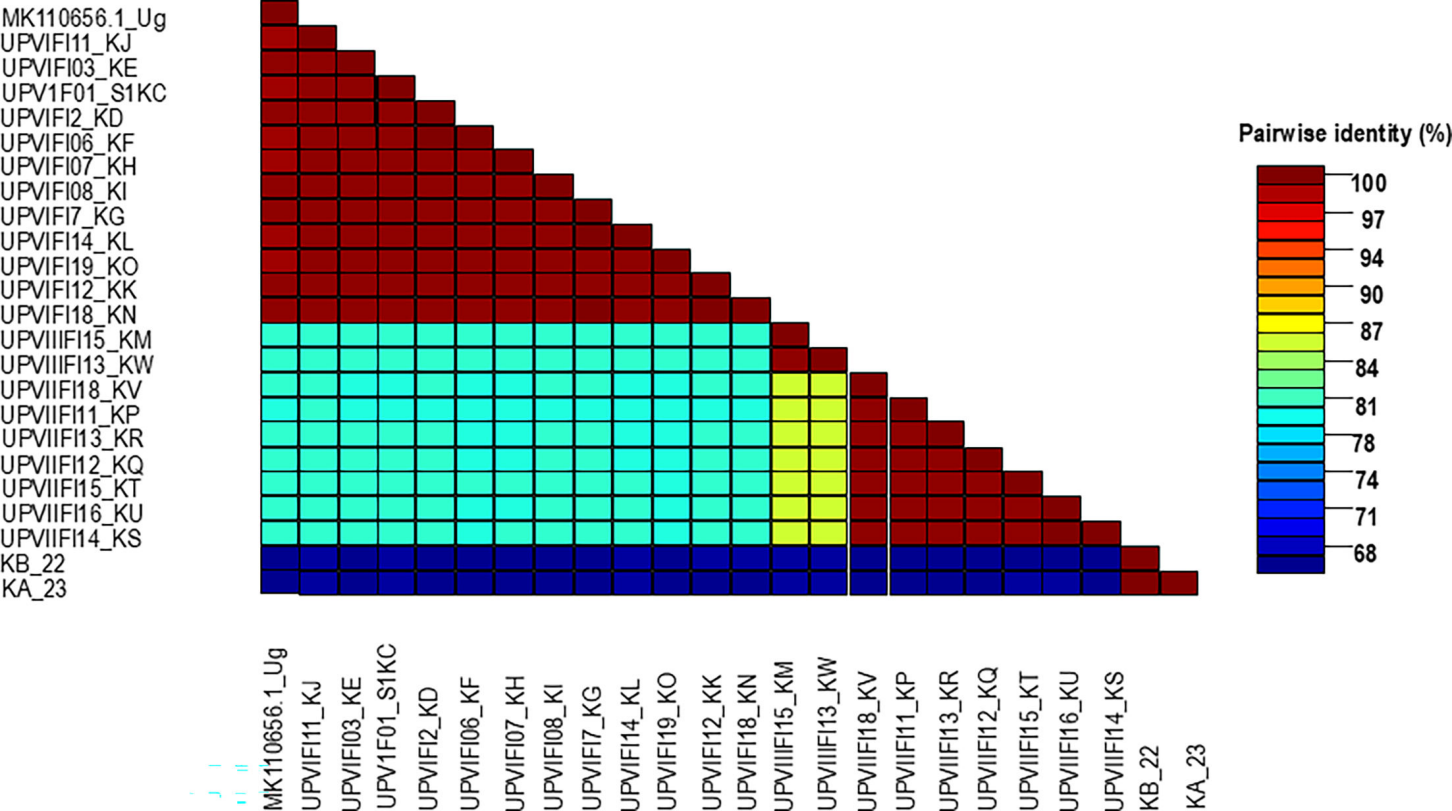
Species demarcation tool interface of the new passiflora virus genome sequences obtained in this study. The color-coded matrix of pairwise identity scores shows species nt pairwise identity scores of the new 21 genomes and the Ugandan isolate currently available in GenBank.

**Fig 3:**
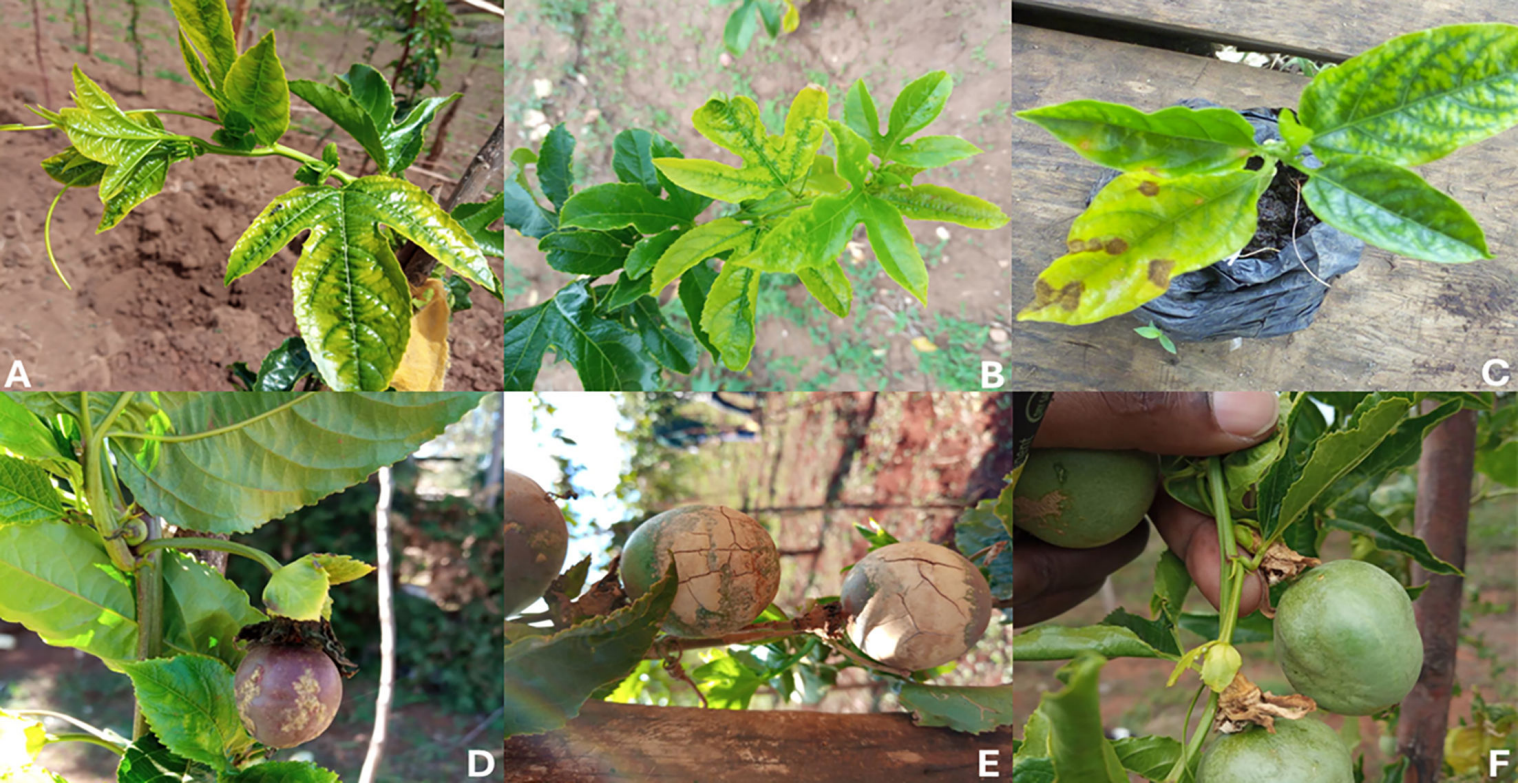
Diversity in passion fruit virus symptoms observed in the field. (**A**) Chlorosis and mottling. (**B**) Severe mosaic on the upper leaves, rugosity, and distortion. (**C**) Nursery seedling with leaf necrosis and mosaic on the lower surface of the leaves. (**D**) Fruit deformation and discolored rind. (**E**) Thick, hard, and woody rind and cracked fruits. (**F**) Deformed fruits.

A total of reads ranging from 1,465,364 to 3,200,244 reads were obtained from the HTS of different samples collected in Kenya’s passion fruit growing regions (Table 2). After *de novo* assembly, the contigs of interest had 4,424–232,782 reads mapping to the virus genome of interest, with an average depth of 61.9–3,159 and a normalized depth of 24.3–1,529. A total of 21 UPV and 2 East Asian passiflora distortion virus (EAPDV) complete genomes were obtained. UPV and EAPDV coinfections were detected in sample 2 and 7 (Fig. 2 and 3). The NCBI and the new UPV genomes were compared and revealed 81%–100% nucleotide (nt) identity. Isolates 1KC, 3KE, 6KF, 7KH, 7KG, and 8KI (Central region) and 11KJ, 12KK, 14Kl, 18KN, 19KO, and 12KD (Rift Valley region) had 99%–100% nt identity match between the isolates (Fig. 2). Isolate KW from Uasin Gishu and isolate KM from Elgeyo Marakwet (Rift Valley region) were identical but shared 86% nt to any other closest isolate, and 81% nt identity match to KO and KN, all from the Rift Valley region. In addition, isolates KW and KM shared 81% nt identity with five isolates from the Central region. The highest UPV genome matched GenBank accession MK110656 from Uganda with 99% nt identity to Rift Valley and Central region isolates, but 81% nt identity to nine isolates from the Rift Valley region. The two new EAPDV sequences (KA and KB) were both detected in Kirinyaga and Murang’a counties in the Central region and had 65%–66% nt identity match to any of the new UPV genomes, but 77.2% nt identity match to the genome of the Japanese isolate PY-AK (Fig. 2). The RT-PCR gel electrophoresis confirmed the presence of UPV 447 (bp) and EAPDV 947 bp coat protein regions. Moreover, UPV variants and two partial passion fruit green spot virus sequences and one partial (passiflora emaravirus) segment RNA1-5 (a new allexivirus and an emaravirus, respectively) were detected. Passiflora virus A was also detected co-infecting with UPV in samples 15 and 22 (Table 2). The complete genomes have been deposited in GenBank with accession numbers MW355818 to MW355840.

**TABLE 2:** Viruses identified in different samples, including the total number of raw reads obtained, length of assembled genome sequences, number of sequence reads mapped, average and normalized sequencing depth

Sample	Raw reads	Virus identified	Contig length	Sequence reads mapped	Average depth	Normalized depth
1_KC	1,545,706	UPV-I	9,684	13,294	183	118.6
2_KA 2_KD	1,509,132	EAPDV	9,638	17,410	232.5	154.1
		UPV-I	9,679	175,623	2,307.	1,529
3_KE	3,200,244	UPV-I	9,670	36,941	516.8	161.5
6_KF	2,547,696	UPV-I	9,650	4,424	61.9	24.3
7_KH 7_KB	1,149,924	UPV-I	9,779	66,140	939.9	817.4
		EAPDV	9,638	11,276	162	141
8_KI	1,465,364	UPV-I	9,874	85,227	1,217	830.7
11_KJ 11_KP	2,578,474	UPV-I	9,669	9,217	117.9	45.8
		UPV-II	9,654	14,188	181.7	70.5
		CABMV (21)	9,846	14,888	185.9	72.1
12_KK 12_KQ	2,706,154	UPV-I^*a*^	9,650	75,058	1,039.6	384.2
		UPV-II^*a*^	9,669	32,348	431.3	159.4
13_KR 13_KW	1,779,280	UPV-II	9,877	81,444	1,151.8	647.4
		UPV-III	9,683	43,291	620.2	348.6
14_KL 14_KS	2,974,246	UPV-I^*a*^	9,629	9,318	131.3	44.1
		UPV-II	9,771	220,180	3,062.3	1,029.6
15_KT 15_KM	1,706,154	UPV-II	9,658	77,940	1,044.6	612.2
		UPV-III	9,667	127,649	1,696.5	994.3
16_KU	1,627,772	UPV-II	9,677	41,050	557	342.3
17_KG	2,400,688	UPV-I	9,651	232,782	3,159	1,315.9
18_KN 18_KV	2,395,822	UPV-I	9,671	185,274	2,619	1,093.3
		UPV-II	9,680	126,554	1,792.6	748
19_KO	1,847,440	UPV-I	9,636	55,234	735.9	398.4

^*a*^ Additional variant of the virus was identified but could not be fully assembled: CABMV, cowpea aphid-borne mosaic virus 2 (passiflora virus A). Samples 4, 5, 9, 10, 20, 21 (incomplete UPV), and 22 (incomplete UPV and passion fruit green spot virus).

### Phylogenetic and recombination analysis

**Fig 4:**
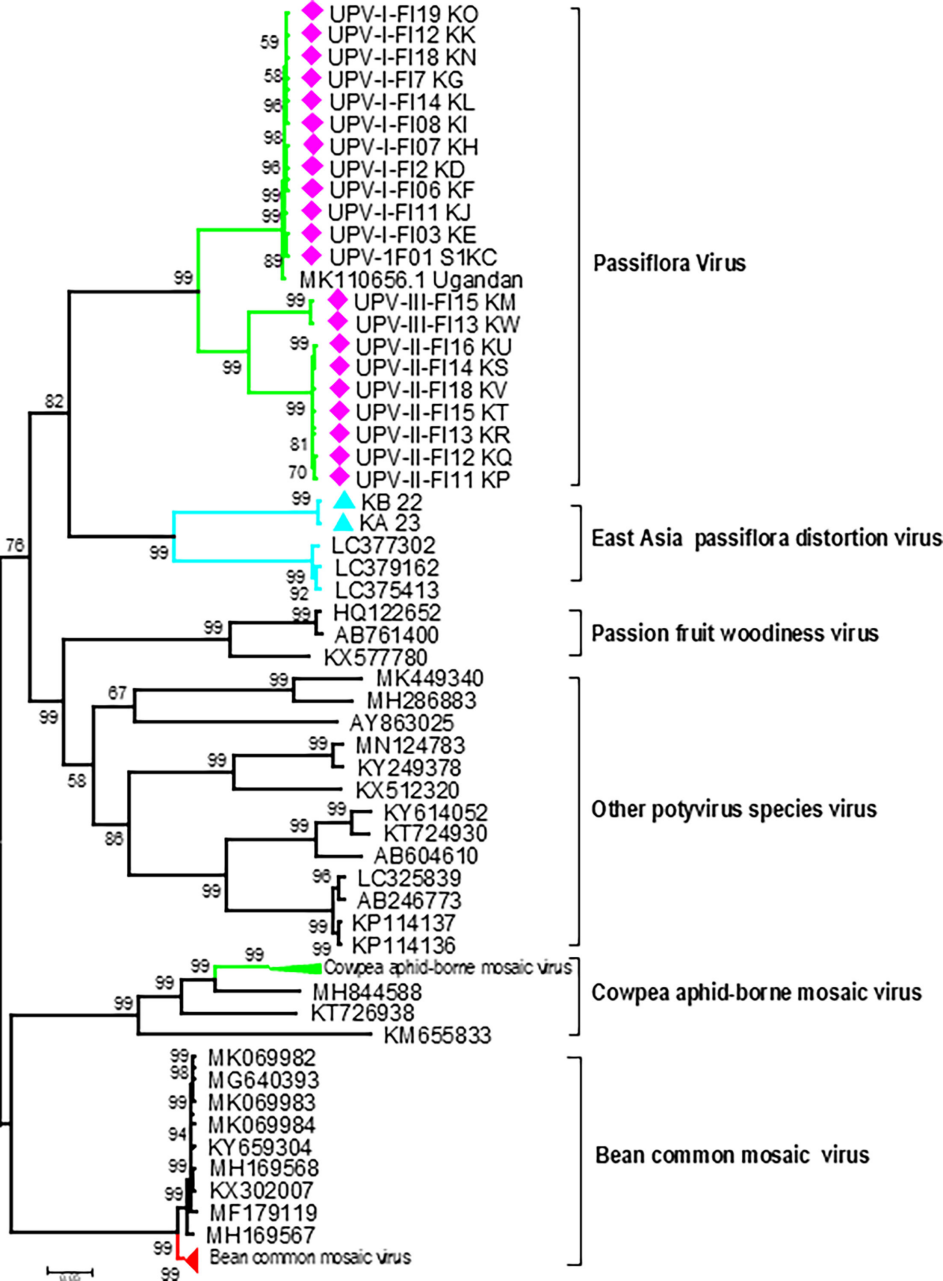
Phylogenetic analysis of 21 new UPV and two EAPDV (KA 23 and KB 22) isolates, along with other closely related potyviruses from GenBank. The tree was generated using MEGA using the maximum likelihood method, and bootstrap values (≥50%) are shown at the nodes. Scale bar indicates evolutionary distance.

When all the new 21 UPV and two EAPDV (KA 23 and KB 22) genomes were compared with other closely related passiflora-associated potyviruses from GenBank, the phylogenetic analysis revealed distinct lineages (Fig. 4). The distinct lineages identified in this study comprised UPV phylogroups I–III, EAPDV, PWV, passion fruit severe mottle virus, bean common mosaic virus, soybean mosaic virus, watermelon mosaic virus, and EAPV. UPV phylogroup I included sequences from the Central and Rift Valley regions of Kenya, along with the UPV-MK110656 isolate from Uganda. In contrast, the minor phylogroups II and III consisted exclusively of UPV sequences from the Rift Valley (Fig. 4). Notably, CABMV and bean common necrosis virus formed a distinct phylogroup, separate from the others. The Kenyan CABMV isolate 11KP (21) clustered closely with eight other CABMV sequences from diverse global regions (Fig. 4). No significant putative recombination breakpoints were detected among the 21 new UPV genomes and the two EAPDV genomes (KA 23 and KB 22) obtained in this study.

### Symptoms

The passion fruit leaf samples exhibited a range of virus-like symptoms, including wrinkling, mosaic patterns, chlorosis, vein clearing, ring spots, and leaf distortion (Fig. 3). However, the expression of symptoms varied notably across different regions. For example, samples from Trans-Nzoia displayed yellow leaf spots, flecks, mosaic-associated mottling, rugosity, and distortion. In Kiambu, mosaic symptoms were observed on both the upper and lower leaf surfaces, with co-infection by CABMV and UPV. Additionally, mosaic symptoms were noted on passion fruit seedlings in Kiambu. Samples from Murang’a exhibited symptoms of thick, hard, woody rinds and cracked fruits, with the presence of UPV and CABMV confirmed. Elgeyo Marakwet samples had severe fruit deformation. In the neighboring region of Uasin Gishu, samples showed pronounced mosaic on the upper leaf surfaces, with the presence of UPV and CABMV co-infection confirmed.

## DISCUSSION

Plant virus metagenomics has the capacity to detect viruses either as single or co-infections and can reveal the presence of novel or unsuspected plant viruses (39, 58). To elucidate the passion fruit virus disease complexity in Kenya, virus surveys were conducted across major passion fruit growing counties. The study revealed the first full genomes of UPV and EAPDV in symptomatic passion fruit farms in Kenya. Passion fruit viruses co-infections may interact either in a synergistic or antagonistic way (59), this remains to be determined in Kenya. Our findings discovered several co-infections such as UPV and EAPDV. The discovery of UPV in major passion fruit growing regions of Kenya, concur with earlier studies reporting the presence of UPV in East Africa (20), and has a close genetic link to the single UPV genome. The UPV and EAPDV sequences grouped separately from the other passion fruit and potyviruses. A distinct lineage was formed when the 21 new UPV and EAPDV genomes were evaluated against other closely related potyviruses that are associated with the genus *Passiflora*. Recombination analysis found no significant putative recombination break points detected when all the new UPV and two EAPDV genomes were analyzed. However, the CABMV had a strong recombination hotspot toward the 5′ UTR region of the virus.

Our results enabled the delineation of three UPV minor phylogroups named I–III. The minor phylogroup I had new sequences obtained from Central, Rift Valley, and Uganda, and the minor phylogroups II and III had all the UPV sequences from Rift Valley only. The new EAPDV was grouped separately from the UPV but together with the other four Japanese EAPDV sequences. All the other potential viral genome sequences associated with passion fruit diseases retrieved from NCBI were grouped separately from the Kenyan sequences. Since the UPV sequence nt identities of the samples from different passion fruit-growing counties in Kenya ranged from 81% to 100%, this supports the conclusion that the current Kenya UPV isolates belong to the same *Potyvirus* species (20, 60). EAPDV sequences had 65%–66% nt identity to the UPV genomes but 77.2% to the closest Japanese genome sequence isolate. Thus, neither the complete genome nor the coat protein (CP) comparisons found any evidence of a close genomic relationship between the new UPV and EAPDV. Our analysis suggested that the observed nt identities shared by the new UPV and EAPDV isolates from Kenya support the classification criteria of the new EAPDV from Kenya as a distinct species within the genus *Potyvirus* as previously found (29).

The presence of UPV genome sequences from Kenya within three minor phylogroups suggests that multiple introductions might have occured in Kenya from adjacent neighboring countries, or vice versa. The previous report of UPV, genome sequence from Uganda, and its high similarity to the Kenyan isolate could suggest that its introduction was most likely from adjacent comtries, either directly or indirectly, or vice versa (61). Similarly, UPV was reported in passion fruit virus-infected samples from Rwanda, showing high homology to the UPV from Uganda based on the CP gene. This could be attributed to the complexity of countries in the East African region geographic positions, such as land borders. There is therefore a need to strengthen quarantine measures due to the nature of the porous borders, which could be playing a key role in the cross-border movement of the viruses via infected planting materials. Further, the Kenyan Western region border with neighboring countries such as Uganda via a porous land crossing, where traders from both countries frequently interact. As such, unregulated trade practices, exemplified by activities such as cyclers or pedestrian trade transporters, may have inadvertently facilitated the dissemination of infected seeds or young seedling along unmonitored routes, then dispersed to the rest of the country, as seen in other parts of the world (61, 62). These reports of UPV infecting passion fruit in Kenya, Uganda, and Rwanda suggest that UPV strains are spreading in East Africa and may be a problem more likely to emerge widely in Africa, thereby posing a threat to the passion fruit industry. Nonetheless, additional sequencing is required to avail more complete genomes from the East African region and other parts of the world. This will uncover UPV and EAPDV global distribution and enhance accurate determination of its origins, evolution, and molecular epidemiology toward improved passion fruit disease management. Furthermore, the availability of these genomes will facilitate the development of sensitive virus diagnostic assays for adoption in virus indexing of nurseries stock in certification programs, as well as supporting diagnostic laboratories that undertake plant health regulation mandate.

Our study proposes that the new currently known EAPDV “East Asian passiflora distortion virus” be appropriately renamed to “passiflora distortion virus (PDV)” to avoid geographical association. UPV was previously detected in Uganda, and a neutral name “passiflora virus” (PV) was proposed (20). The new UPV sequences will equally follow the name PV. Our study has also adopted Latinized numerals in naming the phylogroups of these new sequences and other closely related species. Adopting such neutral virus names deters misleading geographical names such as country names (37, 63). Neutral naming, hereafter such as “PV and PDV,” allows a consideration of the only detected virus sequence that identifies the new virus, without a biased notion associated with the first geographical detection locality as the primary origin of the virus. This is because, in several cases, very limited surveillance and diagnostic activities are conducted prior to such naming being adopted. Consequently, it translates to a lack of broad-spectrum understanding of the etiology of the new virus. For example, the sweet potato virus East African strain was first identified in East Africa, and “East Africa (EA)” was adopted as the strain name (64, 65). The “EA” was the phylogroup distribution originally considered restricted to this virus, leading to the speculation that the EA is the subcenter of this virus diversity and evolution (66, 67). After 13 years, this virus was identified in East Timor, grouping with many other sequences from southern Africa and Southeast Asia, and neutral naming (I) was adopted (68). As such, in the current study, we propose geographically neutral naming such as “PV and PDV,” since these viruses might be circulating in many other parts of the world and are yet to be detected. Notably, due to the increasing world trade in plants and plant products, it is only a matter of time before UPV and PDV are detected in additional countries. Therefore, neutral names such as PV and PDV will minimize the unnecessary implications in virus research, i.e., evolution analysis, viral disease management, stigmatization of a particular region, and potential international trade implications.

Thus, the molecular, serological, and host range studies indicate that the viral disease symptoms on passion fruit in Kenya are caused by a confection of multiple viruses with a limited genetic variation among UPV isolates. Sequences corresponding to normalized read depth between samples varied significantly, indicating differences in host virus titer. The PV occurring in Kenya was isolated from passion fruit leaves showing chlorotic mottle, distortion, thick, hard, and woody cracked fruits, which indicate the presence of PWD in Kenya. These symptoms have been previously found in passion fruit crops in Kenya (34) and neighboring countries, including Uganda (7) and Rwanda (61). Notably, CABMV has also been associated with PWD, which is linked to mosaic, blistering, and distortion on leaves, fruit woodiness, reduction in juice yield, and shorter lifespan (21, 69). Our findings suggest that potential synergistic interactions among PV, CABMV, PDV, emaraviruses, and allexiviruses in influencing symptom development cannot be ruled out. In addition, environmental conditions and host genotypes may significantly contribute to disease expression. Therefore, the distinct symptoms of PWD observed in Kenya are likely influenced by a combination of host and environmental factors, as well as synergistic co-infections involving PV, CABMV, and PDV. We therefore suggest that the new PV and PDV are distinct virus species associated with passion fruit woodiness disease, which is linked to significant economic damages in Brazil, Japan, Taiwan, and East Africa (19, 20, 29, 70). The propagation nature of passion fruit in Kenya include grafting, which leads to the accumulation of viruses (34). Stringent management measures, including virus indexing of planting seedlings at nursery multiplication blocks, are key to preventing further virus dissemination. In addition, epidemiological studies are needed to understand the role of legume crops in spreading CABMV to adjacent passion fruit growing hedges.

Recombination plays a pivotal role in viruses (71). Most plant RNA viruses have high rates of recombination, leading to the emergence of new virus species and their variants, a crucial factor in development of resistance-breaking strains, virulence increase, and change of host range as an adaptation strategy (37, 72, 73). Despite the critical role of the recombination process in RNA viruses, the current new PV and PDV genomes isolated from Kenyan passion fruit crops had no significant evidence of recombination break points. Although the recombination process varies extensively among viruses (71), genetic, biological, and epidemiological factors can affect the probability of virus recombination between different strains or species. This may include genetic homology, existing virus population in the host, co-circulation in the same geographical area, and coinfection rate. The current sequences insufficient genetic diversity (99%–100% nt), may contribute to the lack of recombination. Notably, this large proportion of PV’s insufficient genetic diversity could be attributed to the use of a common seed stock sourced from a multiplication nursery centre before being distributed across the country. Also, the substantial genetic divergence of 34% nt difference between the newly identified PDV and approximately 20% among some new PV isolates is likely to impede the formation of viable recombinant offsprings due to the extent of genomic variation. Importantly, viral recombination is replication dependent; as such, high viral titer has been shown to increase the chances of exchanging genetic strands (74). However, despite the notable symptoms observed in the field samples, it is unclear if the PWD-causing viruses detected in this study existed with an overall high viral load. Future studies are required to decipher the PWD viral load and its role in recombination.

In conclusion, the study reveals the causes of passion fruit viral disease in Kenya, emphasizing the role of mixed viral infections in the manifestation of PWD. This study calls for a shift toward neutral virus naming, such as “PV” and “PDV,” to avoid geographical bias and better reflect the global distribution of these viruses. Our findings underscore the need to strengthen the current in-country nurseries certification programs and intensify pest surveillance activities. Such efforts include stringent quarantine measures, enhanced pest diagnostics on imported plants and plant products, to safeguard Kenya’s horticultural industry from severe viral diseases. Lastly, due to the unpredictable effects of climate change, we recommend additional monitoring activities as an early warning system response to prevent the impact of potential viral vectors.

## Data Availability

The complete genomes have been deposited in NCBI with accession numbers MW355818 to MW355840.
